# Anatomical Ignorance Resulting in Iatrogenic Causes of Human Morbidity

**DOI:** 10.7759/cureus.56480

**Published:** 2024-03-19

**Authors:** Taylor Moglia, Catherine Falkenstein, Finn Rieker, Nang Tun, Mathangi Rajaram-Gilkes

**Affiliations:** 1 Medical Education, Geisinger Commonwealth School of Medicine, Scranton, USA

**Keywords:** flipped classroom, anatomy in clinical years, anatomy in preclinical years, radiology in anatomy, anatomy education

## Abstract

This article discusses how inadequate anatomy education contributes to iatrogenic causes of human morbidity and mortality. Through a review of the relevant literature, high-yield clinical cases were identified in which a lack of sufficient anatomical knowledge contributed to patient morbidity, such as abscess formation and neuropathy as a result of improper intramuscular injections, superior gluteal nerve injuries due to surgical procedures, and misdiagnoses due to physicians’ inability to examine and correlate clinical and radiological findings. The importance of a multimodal learning approach in anatomy education for medical students, which includes the utilization of the cadaveric dissection approach to emphasize spatial understanding, is crucial for the development of competent physicians with a deep-rooted foundational knowledge of anatomy and related concepts, such as physiology, pathology, and radiology. It cannot be understated that anatomy education and a lack of knowledge of anatomy and related concepts may influence iatrogenic causes of human morbidity and mortality. Therefore, all efforts should be made to ensure that students develop a strong foundational anatomy knowledge during their preclinical years.

## Introduction and background

Iatrogenesis is any injury or illness as a result of medical intervention [1]. The word itself is derived from the two Greek words “iatros” and "genesis," meaning physicians and origin, respectively [1]. This can include, but is not limited to, adverse drug effects, medical errors, bedsores, infection, malnutrition, unnecessary procedures, and surgery-related complications [2]. Iatrogenic complications can be the result of negligence, ignorance, inexperience, mistakes, and communication problems [1]. It was estimated in 2005 that the total number of iatrogenic deaths falls just under 800,000, with some research groups calling the American healthcare system a leading cause of death in the United States [2]. In a study of 815 hospitalized patients, 36% experienced some form of iatrogenic complication [3]. However, while tracked at most institutions, iatrogenesis is considered to be underestimated.

A deep-rooted understanding of anatomy is necessary to mold competent physicians, especially surgeons [4]. Hence, establishing effective anatomy teaching methods for medical students, such as the common full-body cadaver dissection, is paramount to the development of well-trained physicians with better patient outcomes [4]. Surveys administered to medical students have shown that anatomy is considered one of the “most essential” pieces of clinical practice and that the understanding of the inter-relationship between anatomy, physiology, pathology, radiology, and clinical sciences is crucial to making diagnoses and developing treatment plans [5]. Additionally, cadaveric dissections allow students to become familiar with anatomical variation and visualize the complexities of underlying structures, which is not entirely reproducible when using technology-based approaches [6]. In summary, inadequate anatomical knowledge due to fewer hours and less access to cadaveric teaching methods poses a significant risk, especially in surgical specialties.

Anatomy is taught during the preclinical curriculum, which has traditionally comprised the first two years of medical school [7]. However, a considerable number of medical schools are shortening the preclinical curriculum to 1.5 years, which has resulted in a shift from in-person learning to the incorporation of online learning methods in preclinical years [7]. According to data from the Liaison Committee on Medical Education, during the 2019-2020 academic year, 6% of schools had a one-year preclinical curriculum, 29% of schools had a 1.5-year preclinical curriculum, and 56% of schools had a two-year preclinical curriculum [8]. Ever since the medical education reform in 2010, anatomy education has been ever-changing [9]. A 2022 study including 145 AAMC-associated allopathic medical schools discovered that most schools reported a major change to their anatomy courses in the past five years, which has resulted in an overall decrease in total course time, integration of anatomy into other courses, and the implementation of a flipped classroom style of teaching for anatomy [9].

With the recent changes in medical education in preclinical years, cadaveric dissections are shifting to a more technology-based learning approach. However, qualitative analyses have demonstrated that human dissection enhances the learning experience by promoting identity formation, self-reflection, and team-building skills [10]. Even with a lack of standardization for anatomy education, there is evidence that medical students benefit from combining multimodal and system-based learning approaches [11]. To optimize learning, it is recommended to incorporate dissection and prosection, interactive media, procedural anatomy, surface and clinical anatomy, and imaging [11].

A 2022 study by Shin et al. analyzed the impact of the COVID-19 pandemic on anatomy education by analyzing curriculum changes prior to and after the pandemic [9]. It was discovered that there was a significant reduction in course time dedicated to hands-on learning and teaching of clinical correlates and radiology due to the pandemic. Additionally, anatomy course directors reported that the decreased interactive learning and a lack of cadaveric dissection negatively impacted the quality of learning for medical students. Schools anticipate the future of anatomy education shifting toward the incorporation of more virtual reality applications and less time in the cadaveric dissection lab [9]. Critically, although these multimodal learning approaches have been deemed effective and favored by both students and faculty members, evidence suggests that these modalities should remain as only a supplement to, and not a replacement of, the necessary component of anatomy education: cadaveric dissections [11,12].

## Review

Methods

**Figure 1:**
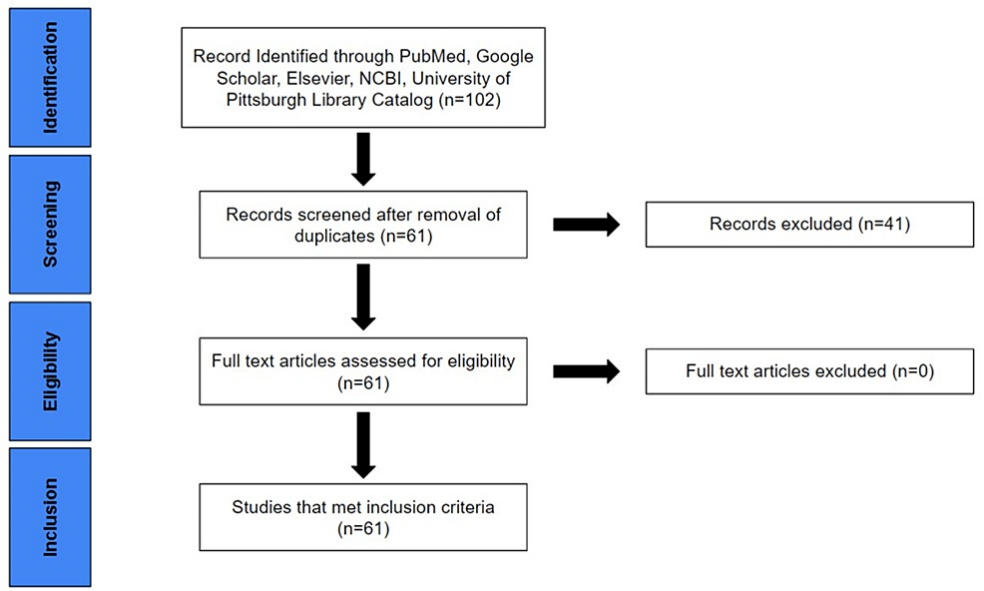
PRISMA flow chart detailing the selection of resources. PRISMA, Preferred Reporting Items for Systematic Reviews and Meta-Analyses

In order to review the current state of undergraduate medical anatomy education and the consequences of anatomical ignorance, a literature search was conducted via PubMed, Google Scholar, Elsevier, NCBI, and the University of Pittsburgh Library Catalog. Search terms included “medical school anatomy,” “anatomical ignorance,” “intramuscular injection iatrogenesis,” “superior gluteal nerve injury,” “unexplained lymphadenopathy misdiagnosis,” “GERD misdiagnosis,” “cholecystitis iatrogenesis,” “chest x-ray misidentification,” “inadequate cardiovascular exam,” “spondylolisthesis misidentification” and “anatomical surgical errors.” Recent literature on iatrogenic causes of morbidity in regard to the above topics were prioritized and redundant articles were excluded. The date of publication was not among the exclusion criteria. Bibliographies were searched for additional relevant articles. The relevant literature was reviewed, and analysis thereof yielded the following original conclusions concerning high-yield anatomical knowledge and anatomy education. The PRISMA (Preferred Reporting Items for Systematic Reviews and Meta-Analyses) flow chart [13] provided in Figure 1 indicates the initial number of records identified from various resources as 102. Information that fit the criteria of the study narrowed the search to 61 articles, which are included in this study.

Incorrect identification of anatomical structures or misinterpretation of radiographs and laboratory results, whether by experienced physicians or medical students, can result in complications in patient care [14]. In a program that monitored iatrogenic complications over the span of five years in the general and vascular surgery departments of a university hospital, it was found that around 40% of iatrogenic complications were accidental, 25% resulted from faulty techniques or non-compliance, 18% were due to drugs, and just more than 10% were attributed to errors, with the latter category accounting for around 20% of moderate or severe complications and more than 33% of severe cases [14]. Hence, it is important not just for medical students and residents but even highly trained specialists to re-visit and re-engage with clinical anatomy. The diagnostic process begins with patients' signs and symptoms, involving physical examination techniques to analyze anatomical causatives. This relies on a precise understanding of surface anatomy and relevant landmarks to investigate concealed internal morphological distortions in structures, organs, limbs, and systems [5]. The decline of physical examination skills in the new generation of physicians is well documented and amendable by a strong foundation in anatomy education. Properly performed and interpreted physical examination findings can save lives through efficient diagnoses, accurate triaging, and effective treatments [15].

The inclination to rationalize iatrogenic errors and a hesitancy to engage in candid discussions impede a substantive examination of this issue. Systematic reduction of iatrogenic complications can be achieved through dedicated examination of root causes, followed by transparency between an institution and its patients, comprehensive anatomy education of medical and paramedical personnel, continuous quality monitoring, and critical reevaluation of medical practices, necessitating the establishment of a robust system for reporting and analyzing each iatrogenic complication [14].

Discussion

The following subsections discuss prevalent complications from the literature review investigating iatrogenesis at least partially attributable to anatomical ignorance. Therefore, these topics represent high-yield areas of improvement within anatomy education and are discussed in the following subcategories.

Intramuscular Injections

While rare in immunocompetent patients, 0.4% of patients develop abscesses, bacteremia, skin necrosis, intra-articular infections, or sepsis as a result of incorrectly placed intramuscular injections, predominantly impacting immunocompromised patients [16]. This commonly performed procedure provides an important mode of drug administration when a quick medication uptake with prolonged effects is required [16,17]. Infections most commonly associated with intramuscular injections include those caused by microbes that normally occupy the skin flora, most notably Staphylococcus aureus, and will usually require surgical drainage and intravascular antibiotics [18,19]. Abscesses also occur if the needle selected is too long and medication is deposited too deeply within the muscle tissue [19].

Although a relatively minor procedure, intramuscular injections require proper aseptic preparation and technique to minimize iatrogenic injury [16]. In addition to infections and abscess formation, peripheral nerve injury has also been described as a common form of iatrogenesis associated with intramuscular injections [17]. Commonly injured nerves include the radial and sciatic nerves due to the commonly utilized muscular regions of the upper extremity and buttock regions, respectively [17,20]. Consequences of damage to these nerves, including any range of minorly painful neuropathy to complete paralysis, can be life-altering and may require surgical intervention. Although incidence and occurrence are decreasing worldwide, it remains a common and avoidable source of iatrogenesis [17]. Anatomical proximity of an injection to a nerve is considered the single most important factor in the degree of damage that can result from an improperly placed needle [21]. A strong foundation in anatomy, especially that which emphasizes nerve location and injury symptoms, will help minimize the incidence of nerve injury and catch early signs of an injury that has occurred.

Superior Gluteal Nerve Complications

In a discussion on anatomical ignorance yielding human morbidity, the significance of the superior gluteal nerve cannot be overstated. In fact, the majority of superior gluteal nerve injuries are iatrogenic, specifically those occurring during surgery [22]. The superior gluteal nerve arises from the posterior branches of the ventral rami of spinal nerves L4 through S1, exiting the pelvis through the greater sciatic foramen, and supplies the gluteus medius, gluteus minimus, and the tensor fasciae latae [22-25]. During total hip arthroplasty (THA), for example, the superior gluteal nerve is at risk when the lateral or anterolateral approaches are utilized [22-25]. The mechanism of injury typically involves dissection of the gluteus medius and retractor placement beyond the approximately 5-cm-wide safe zone proximal to the greater trochanter of the femur [22-25]. Complicating the matter, this safe zone varies between 3 and 7 cm depending on patient height and anatomical variations [22], with some estimates even ranging from 2 to 8 cm [23]. It has been reported that up to 77% of lateral or anterolateral THA patients experience superior gluteal nerve injury, including subclinical injury detected via electromyography [24].

Furthermore, the anterior approach also presents risks to the superior gluteal nerve. The ascending branch of the lateral circumflex femoral artery is ligated, and cadaveric studies have revealed this artery to enter the tensor fasciae latae within 10 mm of superior gluteal nerve branches in 90% of cases; hence, ligation here can cause iatrogenic nerve injury [23]. Ultimately, injury to the superior gluteal nerve can result in paralysis of the gluteus medius and minimus, as well as the tensor fasciae latae, causing abductor weakness and a Trendelenburg gait [22-25]. These complications contribute to the popularity of the posterior approach, in which the superior gluteal nerve is spared but the sciatic nerve is at risk, though to a far lesser degree, with an injury incidence of approximately 1.5% [24]. Nevertheless, ignorance of the neurovascular anatomy of the hip is too common and may be ameliorable through reforms in undergraduate medical anatomy education.

Lymphadenopathy

Lymphadenopathy is a broadly defined clinical scenario as it refers to a lymph node that is either abnormal in size, consistency, or number [26]. The body has around 600 lymph nodes, of which only those in the submandibular, axillary, or inguinal regions can be felt with palpation in a healthy patient. Lymphadenopathy can be classified as localized" with enlargement in one area or "generalized" with enlargement in two or more noncontiguous areas [26]. A clear history and physical examination can identify an easily diagnosable cause such as an upper respiratory infection, pharyngitis, conjunctivitis, insect bites, or recent immunization. If a definitive diagnosis cannot be made based on the history and physical examination alone, both can still provide valuable information when it comes to ordering further diagnostic tests. For example, some underlying causes for lymphadenopathy that require labs, cultures, or even biopsies include viruses such as Epstein-Barr virus or Cytomegalovirus, rheumatoid arthritis, lupus erythematosus, leukemia, serum sickness, sarcoidosis, Kawasaki disease, dermatomyositis, and amyloidosis [26].

Although it is estimated that the prevalence of malignancy in lymphadenopathy may be as low as 1.1% [27], it remains a challenge to identify cases that are secondary to more critical conditions. Some risk factors for malignant conditions include older age, nodules that are firm and fixed in character, presence lasting more than two weeks, and location in the supraclavicular region [27]. A retrospective study conducted in a primary care office found that of 82 patients who presented with unexplained lymphadenopathy and obtained a biopsy, 29 were identified with malignant lymphadenopathies [28]. It was calculated that malignant lymphadenopathies were diagnosed with a prior probability of 1.1% and an after-referral probability of 11%, which supported the recommendation for an effective selection process for further workup for unexplained lymphadenopathy [28]. Thus, an anatomical understanding of the lymphatic system is necessary to connect the varying presentations of lymphadenopathy to possible etiologies, some of which could be life-threatening to patients if missed.

Gastroesophageal Reflux Disease

Ruling out gastroesophageal reflux disease (GERD) when diagnosing upper abdominal pain requires a surprising amount of anatomical knowledge. The pathophysiology of GERD is due to regurgitation of gastric acid into the distal esophagus, producing heartburn [29]. Atypical presentations include dysphagia, esophageal bleeding, coughing, asthma, chronic laryngitis, hoarseness, dental erosion, belching, and bloating [29]. Given its vague symptomatology, rarer conditions are often misdiagnosed as GERD, resulting in iatrogenesis [29-31]. Since specialist-level knowledge of all rare conditions, with GERD among their differential diagnoses, is impractical, physicians should at least possess an understanding of esophageal anatomy sufficient to eliminate GERD from the said differential diagnoses when appropriate. For example, esophageal trachealization, a characteristic endoscopic finding of eosinophilic esophagitis, is not among the symptoms of GERD [29,32]. While a definitive diagnosis of eosinophilic esophagitis is made via histopathological examination, biopsies are not routinely performed and endoscopic findings are often missed [30]. Failure to recognize trachealization on endoscopy constitutes an ignorance of esophageal anatomy with a negative impact on patient outcomes. A multicenter retrospective study found that diagnostic delay of eosinophilic esophagitis was associated with an increase in long-term complications, such as stricture formation, perforation, and bleeding [30]. Yet another example of a rare condition frequently misdiagnosed as GERD is achalasia, with 20-50% of cases initially misdiagnosed [31]. Endoscopic findings characteristic of achalasia and uncharacteristic of GERD include esophageal dilation and “puckering” of the esophageal sphincter [29,31]. Furthermore, a barium esophagram can reveal the classic “bird’s beak” finding of proximal dilation with distal narrowing [31]. Once again, ignorance of the structural manifestations of achalasia compared to normal esophageal anatomy can cause iatrogenesis in the form of complications and continued suffering from an otherwise treatable condition.

Referred Pain

Referred pain refers to pain that may not be localized in the site of origin [33]. Visceral referred pain refers to pain radiating from an affected organ located in the deep body wall tissues to superficial layers [34]. Identifying organs that share sensory innervation is crucial because visceral pain can be significantly affected when one or both of these organs are affected by injury or disease [34]. Hence, an understanding of the anatomy of the nerve pathways and innervations for internal organs is imperative for understanding referred pain. Some cases where knowledge of visceral referred pain is important include cholecystitis, cholelithiasis, renal calculi, and angina.

Cholecystitis is typically an acute condition that involves inflammation of the gallbladder that is not due to the presence of gallstones [35]. A typical presentation of cholecystitis includes acute right upper quadrant pain, as well as a fever and nausea that may be associated with eating. Additionally, right upper quadrant tenderness is seen upon physical examination [35]. Cholecystitis diagnosis by hepatobiliary scintigraphy is the gold standard, although it is primarily diagnosed with ultrasonography, which may not provide a definitive diagnosis. Once diagnosed, a procedure such as a laparoscopic cholecystectomy or open cholecystectomy may be performed to remove the gallbladder [35]. A review article that analyzed cases of acute cholecystitis from October 2005 to April 2010 found 14 cases of misdiagnosis, with 21% being overcalls and 79% being undercalls [36]. Additionally, 57% of misdiagnoses involved ultrasound diagnosis, and 43% involved computed tomography (CT) diagnosis [36]. Similarly, cholelithiasis is a condition that involves inflammation of the gallbladder that is due to the presence of gallstones [37]. Since only one-third of patients present symptomatically, most cases of cholelithiasis are found incidentally on imaging [38]. Because cholelithiasis can present with similar symptoms and includes similar diagnostic methodologies and management as cholecystitis [39], it is important to understand symptom presentation and radiographic findings to make a clinical diagnosis.

Renal calculi, more commonly known as kidney stones, were the ninth most common cause of emergency visits in 2018 and affect around 7-8% of women and 11-16% of men before 70 years of age [40]. In addition to hematuria and localized pain to the abdomen and flank, patients will often report referred pain along the T10 to S4 dermatomes commonly experienced in the groin, testicle, and labial areas [40,41]. Patients may also exhibit constitutional symptoms including fever, chills, and fatigue. Renal calculi can quickly progress to sepsis and multi-organ failure if untreated, underscoring the importance of properly interpreting referred pain in patients with kidney stones so that they can be accurately and efficiently treated [42].

Angina pectoris refers to the experience of retrosternal chest pain, pressure, or discomfort that may be exacerbated by exertion or emotional stress [43]. While angina may be relieved by rest and nitroglycerin, which would be categorized as stable angina, angina not relieved by these factors is categorized as unstable angina. Unstable angina may be a predictor for cardiac events and can lead to serious morbidity if not properly identified and treated [43]. Angina is caused by an imbalance between oxygen supply and demand in the heart. Most commonly, this can be associated with coronary artery disease where there is an atherosclerotic plaque narrowing the lumen of the blood vessel supplying oxygen and nutrients to the heart [43]. With nearly 10 million adults in the USA experiencing stable angina, the first-line diagnostic test with a high sensitivity and comparable specificity is coronary CT angiography [44]. Thus, ensuring a thorough diagnostic workup for any type of chest pain is crucial to avoid misdiagnoses of severe cardiac events, such as an acute myocardial infarction or an aortic dissection.

Chest X-Rays

Accurate interpretation of chest radiographs is valuable because it is noninvasive, cost-effective, and easily obtained. Identifying diseases or abnormalities at an early stage can lead to more effective and less invasive treatment options. Patients may present with a constellation of symptoms that align with a physician’s differential diagnosis, making imaging the next best step to either support or refute the diagnosis. However, perceptual errors in certain anatomical regions often occur in radiographs, called “blind spots” [45]. One of the cases where failure to recognize key findings on X-rays is a pneumothorax, which commonly presents with acute dyspnea and pleuritic chest pain. When a patient is placed supine, pleural air collects in the subpulmonic pleural space rather than the apex, where it is usually seen in upright patients [45]. Hence, the reader must examine the lung bases in trauma patients or those who are suspected to have pneumothorax [45]. A subtle pneumothorax is often missed because it would appear as a “deep sulcus sign,” with a deep radiolucent costophrenic sulcus in supine film [45]. It is reasonable to conclude that analysis of radiographs or any other imaging modality is not static but incredibly complex. Although X-rays are two-dimensional, one needs three-dimensional knowledge of various anatomical structures and the changes that occur within the body in a pathological state. Having this spatial awareness would enable the physician to identify the dynamic ways the diseased state would appear on imaging.

In another study that surveyed interns on their experience learning radiology in medical school, 52% reported that the education was not sufficient to prepare them upon graduating [46]. A randomized trial examined 100 senior medical students who had a medical imaging learning pathway incorporated into their curriculum [46]. They spent a minimum of 8 hours learning radiology, as well as adaptive learning tutorials on clinical use and imaging interpretations. The conclusion of the study found that students in the clinical phase had greater learning benefits than those in the pre-clinical phase, and the former completed the tasks in less time [46]. The sensitivity in finding pathologies in chest radiographs was between 20% and 65% in one emergency department [47]. General surgeons performed inferiorly to radiologists, by missing 37% of chest/abdominal radiographs [47]. A randomized study was performed with 61 anesthesiologists, attendings, and residents, and a control group of eight radiology residents [48]. They were shown a series of 10 chest radiographs, a clinical scenario, and a set time limit. The results showed that the most commonly missed diagnoses by participants were pneumothorax, free air under the diaphragm, bronchial perforation from a nasogastric (NG) tube, right mainstem intubation, superior vena cava perforation from a central venous catheter, normal film, negative pressure pulmonary edema, left lower lobe collapse, pulmonary infarction, and tension pneumothorax [48]. The findings illustrated the deficiency of anesthesiologists in interpreting chest radiographs and that they depend heavily on radiologists for final interpretation [48]. It was also found that the scores of attending versus resident physicians were not significantly different [48]. This research strengthens the need for further training in anatomy in medical school and during residency to improve diagnostic skills, which are crucial in early disease detection. These studies underscore the ongoing need for improved medical imaging education during medical school and the essential role of deep anatomical knowledge in enhancing diagnostic skills among clinicians.

Cardiovascular Physical Examination

Correctly performed cardiovascular physical examinations are critical in the evaluation of patients with cardiac symptoms but can be complex and multifaceted [15]. An emphasis is placed on anatomical surface landmarks to draw conclusions about the underlying anatomy and possible pathology that causes symptoms such as peripheral edema, dyspnea, cough, wheezing, and chest pain [15]. When combined with a detailed history, a thorough and properly interpreted physical examination can identify nearly 80% of cardiac diseases [15]. A full evaluation of peripheral pulses includes palpation of the radial, brachial, femoral, popliteal, posterior tibial, and dorsalis pedis arteries, and will usually characterize the rate, rhythm, intensity, and symmetry [49]. Failure to palpate peripheral pulses on any level could result in missed findings that could point the physician toward pathologies including peripheral arterial disease, vasculitis, congenital abnormalities, thrombosis, vasospasm, Takayasu arteritis, coarctation of the aorta, and even tumors that impinge on any level of the vasculature [49]. In patients with cardiovascular symptoms, proper auscultation of heart and lung sounds is critical in making correct diagnoses and ruling out disease, when appropriate. Auscultation of lung fields in patients with the disease may reveal, among others, crackles, wheezes, and pleural rubs that may indicate the presence of fluid, obstruction, or infection. Lack of, or diminished, lung sounds could indicate a pneumothorax or congenital disease. Auscultation of heart sounds gives valuable information regarding the health of heart valves, chambers, and walls while also providing insight into the timing within the cardiac cycle that the lesion may be affecting [50].

It is necessary to have a strong foundation in anatomy and pathophysiology to properly place a stethoscope and interpret the sound findings [51]. Auscultation and palpation remain invaluable pieces of a thorough physical examination that, when done properly and when used in tandem with diagnostic testing and imaging, can guide correct diagnoses and treatment. They are quick and easy to perform, inexpensive, noninvasive, safe, and one of the oldest diagnostic techniques, but they require knowledge of surface and bony landmarks, particularly the sternal notch and ribs, which allow for proper palpation and placement of a stethoscope [51]. Failure to identify these structures or inadequate knowledge of the underlying anatomy can lead to false or missed diagnoses that could have fatal consequences for patients.

Spondylolisthesis

Spondylolisthesis refers to the displacement of a vertebral body in reference to bordering vertebral bodies [52]. The degree of slippage is described by a grading system that measures the amount of vertebral slippage related to the caudal vertebrae. Grade I refers to less than 25% vertebral slippage, grade II refers to 25-50% vertebral slippage, grade III refers to 51-75% vertebral slippage, and grade IV refers to 76-100% vertebral slippage [52]. Spondylolisthesis can further be categorized based on etiology into five types [53]. Type I is congenital, with the fifth lumbar vertebrae (L5) slipping anteriorly to the first sacral vertebrae (S1). Type II is isthmic and caused by stress fractures most commonly in children. Type III is degenerative and occurs as a result of increased stress on the spine, and is most common in older adults. Type IV is traumatic and caused typically by an acute injury. Lastly, type V is a pathologic fracture, which is merely used as a descriptor and does not account for progression or severity [53].

The severity of the slippage in spondylolisthesis is determined by diagnostic imaging, most commonly X-ray or magnetic resonance imaging (MRI). Although X-rays are more commonly used, both can serve as valuable tools when determining patient prognosis. X-rays, which are typically taken when the patient is weight-bearing, have been found to be useful for determining the presence and the grading of spondylolisthesis, whereas MRIs, which are typically taken with the patient lying supine, have been found useful to assess for nerve or spinal cord compression [54]. Furthermore, treatment can be either operative or nonoperative depending on the prognosis and individual patient risks such as age. Nonsurgical management includes limited physical activity, non-steroidal anti-inflammatory drugs (NSAIDs), and physical therapy, whereas surgical management includes a surgical decompression procedure, a fusion procedure, or a decompression and fusion combination procedure [55].

Spondylolisthesis cases are challenging since posterior surgery is complicated by unclear anatomical landmarks, adhesion of scar tissue to muscle and dural structures, and difficulty accessing the intervertebral discs themselves [56]. However, there is promise that an anterior lumbar interbody fusion procedure provides further stabilization and avoids reentry of the spinal canal for revision fusion procedures [56]. Additionally, in procedures such as laminectomies, iatrogenic spondylolisthesis can develop as a postoperative complication or even cause patients to have increased risks for instability [57]. One review found that 10 out of 105 patients who underwent a laminectomy procedure developed iatrogenic spondylolisthesis as a postoperative complication at the same level the laminectomy procedure was performed [57]. In summary, interpreting imaging findings is crucial for the diagnosis of spondylolisthesis, and surgery remains a common treatment modality, A strong understanding of normal vertebral anatomy is imperative for successful patient outcomes.

Anatomical Variations

General anatomical ignorance yields ignorance of the prevalence and significance of specific anatomical variations. While the true proportion of medical errors attributable to anatomical ignorance may be obscured by misclassification and underreporting [2], analyses of surgical malpractice claims have found difficult or abnormal anatomy to be a contributing factor in 13% and 25% of cases [58,59], which is in line with other estimates [60].

One of the most significant examples of commonly variable anatomy is in the operative field of laparoscopic cholecystectomy. Proper identification of the hepatocystic and Calot’s triangles, and the anatomy within and around them, is crucial for the safe execution of the procedure [60,61]. Normally, a single cystic artery branches off of the right hepatic artery at the superior border of the cystic triangle, posterior to the common hepatic duct [60]; however, variations in this pattern exist [61], with an estimated prevalence of more than 10% [62]. Similarly, the cystic duct, which normally branches off of the common bile duct, marking the inferior vertex of the hepatocystic and Calot’s triangles, exhibits variable anatomy in at least 4% of patients [61,62]. Ignorance of these anatomical variations can result in complications such as biliary or vascular injuries [61,62]. One analysis of surgical malpractice claims found laparoscopic cholecystectomies to account for 7% of all cases, with an emphasis on common bile duct injuries [58].

The same study found that hernia repairs constituted 3% of the malpractice claims [58]. The inguinal hernia repair specifically is another procedure that is complicated by anatomical variations, leading to iatrogenesis [60,61]. It has been reported that up to 80% of patients possess anatomical variations affecting sensory innervation from the ilioinguinal and ilio-hypogastric nerves [61]. Damage to and compression of these nerves contributes to more than 30% of herniorrhaphy patients suffering from persistent postoperative pain [61], not all of whom file malpractice claims. Ultimately, the prevalence of anatomical variations is yet another reason to strengthen anatomy education in medical schools and beyond.

## Conclusions

Due to shortening of the preclinical phase of medical education, the classical approach of anatomy instruction has been compressed to fit into a restricted time frame with several areas of the human body left unexplored by dissection. Understanding the three-dimensional organization of various anatomical structures and their respective place within the human body plays a pivotal role in the interpretation of the spatial relationship of the various organs and organ systems as well as the clinical implications. Irrespective of the curricular plan used by individual institutions, it is our recommendation that spending adequate time on the understanding of human anatomy via dissection and practical assessments, in addition to interpretation of imaging modalities and surface anatomy, will help solidify the knowledge.

## References

[1] Peer RF, Shabir N (2018). Iatrogenesis: a review on nature, extent, and distribution of healthcare hazards. J Family Med Prim Care.

[2] Null G, Dean C, Feldman M, Rasio D, Smith D (2005). Death by Medicine.

[3] Kohn LT, Corrigan JM, Donaldson MS, editors editors (2000). To Err is Human: Building a Safer Health System. https://www.ncbi.nlm.nih.gov/books/NBK225187/.

[4] Estai M, Bunt S (2016). Best teaching practices in anatomy education: a critical review. Ann Anat.

[5] Singh R, Yadav N, Pandey M, Jones DG (2022). Is inadequate anatomical knowledge on the part of physicians hazardous for successful clinical practice?. Surg Radiol Anat.

[6] Turney BW (2007). Anatomy in a modern medical curriculum. Ann R Coll Surg Engl.

[7] Verma N, Yui JC, Record JD, Hueppchen NA, Naik RP (2024). The changing landscape of the preclinical medical school curriculum: results from a Nationwide Survey of United States Medical School Curriculum Deans. Am J Med.

[8] Association of American Medical Colleges (1994). AAMC Directory of American Medical Education. Association of American Medical Colleges.

[9] Shin M, Prasad A, Sabo G, Macnow AS, Sheth NP, Cross MB, Premkumar A (2022). Anatomy education in US medical schools: before, during, and beyond COVID-19. BMC Med Educ.

[10] Parker E, Randall V (2021). Learning beyond the basics of cadaveric dissection: a qualitative analysis of non-academic learning in anatomy education. Med Sci Educ.

[11] Sugand K, Abrahams P, Khurana A (2010). The anatomy of anatomy: a review for its modernization. Anat Sci Educ.

[12] Papa V, Varotto E, Galli M, Vaccarezza M, Galassi FM (2022). One year of anatomy teaching and learning in the outbreak: Has the Covid-19 pandemic marked the end of a century-old practice? A systematic review. Anat Sci Educ.

[13] Moher D, Liberati A, Tetzlaff J, Altman DG (2010). Preferred reporting items for systematic reviews and meta-analyses: the PRISMA statement. Int J Surg.

[14] Adar R, Bass A, Walden R (1982). Iatrogenic complications in surgery. Five years' experience in general and vascular surgery in a University Hospital. Ann Surg.

[15] Malik MB, Goyal A (2024). Cardiac Exam. StatPearls [Internet].

[16] Sambandam SN, Rohinikumar GJ, Gul A, Mounasamy V (2016). Intramuscular Injection Abscess Due to VRSA: A New Health Care Challenge. Arch Bone Jt Surg.

[17] Park CW, Cho WC, Son BC (2019). Iatrogenic injury to the sciatic nerve due to intramuscular injection: a case report. Korean J Neurotrauma.

[18] Moran GJ, Krishnadasan A, Gorwitz RJ, Fosheim GE, McDougal LK, Carey RB, Talan DA; EMERGEncy ID Net Study Group (2006). Methicillin-resistant S. aureus infections among patients in the emergency department. N Engl J Med.

[19] Velissaris D, Matzaroglou C, Kalogeropoulou C, Karamouzos V, Filos K, Karanikolas M (2009). Sepsis requiring intensive care following intramuscular injections: two case reports. Cases J.

[20] Esquenazi Y, Park SH, Kline DG, Kim DH (2016). Surgical management and outcome of iatrogenic radial nerve injection injuries. Clin Neurol Neurosurg.

[21] Kline DG, Kim D, Midha R, Harsh C, Tiel R (1998). Management and results of sciatic nerve injuries: a 24-year experience. J Neurosurg.

[22] Pinho AR, Leite MJ, Lixa J (2023). Superior gluteal nerve anatomy and its injuries: aiming for a more secure surgical approach of the pelvic region. Diagnostics (Basel).

[23] Grob K, Manestar M, Ackland T, Filgueira L, Kuster MS (2015). Potential risk to the superior gluteal nerve during the anterior approach to the hip joint: an anatomical study. J Bone Joint Surg Am.

[24] Hasija R, Kelly JJ, Shah NV, Newman JM, Chan JJ, Robinson J, Maheshwari AV (2018). Nerve injuries associated with total hip arthroplasty. J Clin Orthop Trauma.

[25] Eksioglu F, Uslu M, Gudemez E, Atik OS, Tekdemir I (2003). Reliability of the safe area for the superior gluteal nerve. Clin Orthop Relat Res.

[26] Ferrer R (1998). Lymphadenopathy: differential diagnosis and evaluation. Am Fam Physician.

[27] Bazemore AW, Smucker DR (2002). Lymphadenopathy and malignancy. Am Fam Physician.

[28] Fijten GH, Blijham GH (1988). Unexplained lymphadenopathy in family practice. An evaluation of the probability of malignant causes and the effectiveness of physicians' workup. J Fam Pract.

[29] Maret-Ouda J, Markar SR, Lagergren J (2020). Gastroesophageal reflux disease: a review. JAMA.

[30] Lenti MV, Savarino E, Mauro A (2021). Diagnostic delay and misdiagnosis in eosinophilic oesophagitis. Dig Liver Dis.

[31] Richter JE (2011). The diagnosis and misdiagnosis of Achalasia: it does not have to be so difficult. Clin Gastroenterol Hepatol.

[32] Al-Hussaini AA, Semaan T, El Hag IA (2009). Esophageal trachealization: a feature of eosinophilic esophagitis. Saudi J Gastroenterol.

[33] Procacci P, Maresca M (1999). Referred pain from somatic and visceral structures. Curr Rev Pain.

[34] Pacheco-Carroza EA (2021). Visceral pain, mechanisms, and implications in musculoskeletal clinical practice. Med Hypotheses.

[35] Gallaher JR, Charles A (2022). Acute cholecystitis: a review. JAMA.

[36] Brook OR, Kane RA, Tyagi G, Siewert B, Kruskal JB (2011). Lessons learned from quality assurance: errors in the diagnosis of acute cholecystitis on ultrasound and CT. AJR Am J Roentgenol.

[37] Reshetnyak VI (2012). Concept of the pathogenesis and treatment of cholelithiasis. World J Hepatol.

[38] Ross M, Brown M, McLaughlin K (2011). Emergency physician-performed ultrasound to diagnose cholelithiasis: a systematic review. Acad Emerg Med.

[39] Schirmer BD, Winters KL, Edlich RF (2005). Cholelithiasis and cholecystitis. J Long Term Eff Med Implants.

[40] Favus MJ, Feingold KR (2000). Kidney stone emergencies. Endotext [Internet].

[41] Leslie SW, Sajjad H, Murphy PB (2024). Renal calculi. StatPearls [Internet].

[42] Portis AJ, Sundaram CP (2001). Diagnosis and initial management of kidney stones. Am Fam Physician.

[43] Kloner RA, Chaitman B (2017). Angina and its management. J Cardiovasc Pharmacol Ther.

[44] Joshi PH, de Lemos JA (2021). Diagnosis and management of stable angina: a review. JAMA.

[45] Ropp A, Waite S, Reede D, Patel J (2015). Did I miss that: subtle and commonly missed findings on chest radiographs. Curr Probl Diagn Radiol.

[46] Ayesa SL, Katelaris AG, Brennan PC, Grieve SM (2021). Medical imaging education opportunities for junior doctors and non-radiologist clinicians: a review. J Med Imaging Radiat Oncol.

[47] Eid JJ, Macedo FI, Negussie E, Mittal VK (2017). Assessing surgical residents' imaging interpretation skills. Am J Surg.

[48] Kaufman B, Dhar P, O'Neill DK, Leitman B, Fermon CM, Wahlander SB, Sutin KM (2001). Chest radiograph interpretation skills of anesthesiologists. J Cardiothorac Vasc Anesth.

[49] Zimmerman B, Williams D (2024). Peripheral pulse. StatPearls [Internet].

[50] Dornbush S, Turnquest AE (2024). Physiology, heart sounds. StatPearls [Internet].

[51] Sarkar M, Madabhavi I, Niranjan N, Dogra M (2015). Auscultation of the respiratory system. Ann Thorac Med.

[52] Vanti C, Ferrari S, Guccione AA, Pillastrini P (2021). Lumbar spondylolisthesis: STATE of the art on assessment and conservative treatment. Arch Physiother.

[53] Wiltse LL, Newman PH, Macnab I (1976). Classification of spondylolisis and spondylolisthesis. Clin Orthop Relat Res.

[54] Alvi MA, Sebai A, Yolcu Y, Wahood W, Elder BD, Kaufmann T, Bydon M (2020). Assessing the differences in measurement of degree of spondylolisthesis between supine MRI and erect X-Ray: an institutional analysis of 255 cases. Oper Neurosurg (Hagerstown).

[55] Samuel AM, Moore HG, Cunningham ME (2017). Treatment for degenerative lumbar spondylolisthesis: current concepts and new evidence. Curr Rev Musculoskelet Med.

[56] König MA, Ebrahimi FV, Nitulescu A, Behrbalk E, Boszczyk BM (2013). Early results of stand-alone anterior lumbar interbody fusion in iatrogenic spondylolisthesis patients. Eur Spine J.

[57] Ramhmdani S, Xia Y, Xu R, Kosztowski T, Sciubba D, Witham T, Bydon A (2018). Iatrogenic spondylolisthesis following open lumbar laminectomy: case series and review of the literature. World Neurosurg.

[58] Rogers SO Jr, Gawande AA, Kwaan M, Puopolo AL, Yoon C, Brennan TA, Studdert DM (2006). Analysis of surgical errors in closed malpractice claims at 4 liability insurers. Surgery.

[59] Regenbogen SE, Greenberg CC, Studdert DM, Lipsitz SR, Zinner MJ, Gawande AA (2007). Patterns of technical error among surgical malpractice claims: an analysis of strategies to prevent injury to surgical patients. Ann Surg.

[60] Alraddadi A (2021). Literature review of anatomical variations: clinical significance, identification approach, and teaching strategies. Cureus.

[61] Kowalczyk KA, Majewski A (2021). Analysis of surgical errors associated with anatomical variations clinically relevant in general surgery. Review of the literature. Transl Res Anat.

[62] Porzionato A, Macchi V, Stecco C, Boscolo-Berto R, Loukas M, Tubbs RS, De Caro R (2022). Clinical anatomy and medical malpractice-a narrative review with methodological implications. Healthcare (Basel).

